# Preparation of Soft Embalmed Cadavers by the Modified Thiel Embalming Technique for Surgical Skill Training and Development of a Universal Quantitative Scoring System to Assess the Suitability of Soft Embalmed Cadavers for Such Training Purposes

**DOI:** 10.7759/cureus.43991

**Published:** 2023-08-23

**Authors:** Amrit G, Satyashree Ray, Soumyashree Mohapatra

**Affiliations:** 1 Orthopaedics, Srirama Chandra Bhanja (SCB) Medical College, Cuttack, IND; 2 Anatomy, Srirama Chandra Bhanja (SCB) Medical College, Cuttack, IND; 3 Microbiology, Srirama Chandra Bhanja (SCB) Medical College, Cuttack, IND

**Keywords:** soft embalming cadaver, formalin, surgical training, skill laboratories, cadaver study

## Abstract

Background

Cadaver dissection plays an important role in learning anatomy. A surgeon must have a thorough knowledge of anatomy of the operating region to perform safe surgery. Skill laboratories give opportunities to surgeons to practice on cadavers before venturing onto real patients. The most common method of cadaver preservation is through formalin fixation. In the process of fixation, formalin destroys the tissue characteristics and also has issues such as smell, eye irritation, hardening of tissue, and risk of carcinogenesis. The Thiel embalming technique and its modifications were developed to address those issues. Our primary objective was to find the benefits of soft embalmed cadavers over formalin-fixed bodies and, secondly, to find out microbial flora in soft embalmed cadavers.

Study design

This is a basic study.

Methods

Four cadavers were prepared for the soft embalming purpose for our workshop for surgeons on spine fixation. Due to unavailability, we replaced 4-chloro-3-methylphenol 1% with phenol 1%. The bodies were preserved in refrigerators at 4^°^C before being used for the workshop purpose. The delegates and faculties were given a questionnaire to assess their experience of the cadavers in terms of odor, irritation, tissue characteristics, joint mobility, and imaging characteristics. The results were calculated using statistical analysis. Swabs were taken from a few of the cadavers for culture to find the organisms.

Results

There were 14 questions in the questionnaire, and the data collected were divided into two groups, faculties, and delegates. JASP software was used to analyze the data. The questions addressed various aspects of cadavers such as color, odor, tissue pliability, joint flexibility, imaging characteristics, mucosal irritation, and earlier experience in working with cadavers. Cronbach α was used to find the correlation between the various characteristics analyzed. The authors intend to name the domains being measured: surgical suitability (scores of items 8 to 12), imaging suitability (scores of items 5 and 6), and smell score (scores of items 5 and 6). It can be a guide to constructing and refining a better quantitative scale to measure the “quality of soft-embalmed cadavers for surgical training.”

Conclusions

Skill laboratories give opportunities to young surgeons and trainees to learn and improve their skills before applying them to real patients. This was our first attempt to develop soft embalmed cadavers at our center and our state. We used the parent solution with some variations as per the availability of chemicals at our place and found that the features of the preserved cadavers were good and well-suited to address our purpose. Therefore, with some variations in the parent formulations, centers situated in remote and less developed places can formulate their own solution to develop soft embalmed cadavers and establish cadaver skill laboratories. This will benefit the local surgeons and trainees. The authors tried to develop a few domains through statistical analysis, which can be used to assess and compare the quality of cadavers prepared at various centers.

## Introduction

Practice makes a person perfect. This statement holds during medical curricula, especially in surgical specialties. Practicing on an artificial mannequin or simulation followed by practice on cadavers makes the transition to operating on patients smoother, more practical, and safer. The practice of cadaver dissection dates to the Ancient Greek period during the third century BC [1,2]. The dark age of the 14th century due to religious beliefs ended with the European Renaissance at the beginning of the 15th century, which revived cadaver dissection. From the 16th century onward, anatomical theaters were set up across Europe for the public. As the interest in dissection grew, the demand for cadavers rose exponentially. Initially, the bodies of executed criminals who were guilty of heinous crimes in accordance with the law of the times in Europe were used for dissection. Gradually the demand for bodies grew and those executed criminals could not meet it. Therefore, people, including famous anatomists, resorted to infamous and illegal ways to obtain bodies. Some body snatchers and murderers sold dead bodies to anatomists. The Murder Act of 1752 made the body of executed criminals available for dissection. Later, the Anatomy Act of 1832 made it legal to use unclaimed bodies for dissection. The Uniform Anatomical Gift Act (UAGA) came into force in the United States in 1968, which made the donor’s body his/her property so that their dying wish for body donation superseded the will of their next of kin. The Human Tissue Act of 2004 established a hierarchy among the relations of the deceased to supply consent for body donation. India has no body donation law, and the program is run here as per the 1949 Anatomy Act and its amendments. In 2012, the Odisha Anatomy (Amendment) Bill was passed, and other states also introduced a similar bill to ease body donation. The preservation of bodies has a long history, starting from the era of mummification and cryopreservation to the modern era of embalming. Embalm is defined in the Merriam-Webster dictionary as “to treat (a body) to protect.” Any embalming fluid consists of a preservative or fixative, disinfectant, modifying agent, perfuming agent, dyes, and diluent. Formalin was the first chemical that was used to embalm after William Harvey discovered the circulatory system. The embalming techniques were widely divided into formalin-based and non-formalin based, which are again divided into hard and soft fixed. None of the non-formalin-based techniques can be defined as hard-fixed techniques. The Thiel embalming technique, which was started in the year 1992, gained wide popularity due to its advantage of preserving the normal tissue quality. Formalin-fixed cadavers are hard and discolored and do not mimic the natural tissue characteristics. The formalin vapor is also considered carcinogenic. The Thiel embalming technique uses a small amount of formalin along with sodium nitrate, potassium nitrate, and sodium sulfide. The chemicals used in the Thiel embalming are considered poisonous, very flammable, explosive, extremely hazardous to health, and dangerous to the environment. To reduce the formation of furans and dioxins, Thiel cadavers must be cremated at high a temperature, which is considered a disadvantage. In our study, we have prepared four soft embalmed cadavers, used them for the workshop, and recorded the opinions of the participants based on a questionnaire for comparative analysis.

## Materials and methods

In the present scenario in India, the requirement of cadavers for dissection and academic studies is mainly met by unclaimed bodies available at various medical colleges and hospitals. The awareness of voluntary body donation is gradually becoming widespread, and, after a few years, they will serve as bodies for educational purposes. The unclaimed bodies of the destitute are normally stored in a cool room for two to three days before the legal processes are carried out by police. If no one turns up to claim the dead bodies, the bodies are either handed over to NGOs for their final rites or to the anatomy department of the medical college attached for preservation by embalming for future studies. Normally, the bodies are embalmed with formalin for hard fixed embalming. We needed soft embalmed bodies for the cadaver workshop. Therefore, it was planned to have at least four soft embalmed bodies for the workshop. One formalin-fixed hard embalmed body was used to show the navigation system.

Walter Thiel developed a low odor embalming technique after working for more than 30 years with around 977 complete cadavers, cadavers after autopsy, and in vitro series of fresh beef [3]. His technique of embalming the bodies used 4-chloro-3-methylphenol, as well as various salts for fixation, boric acid for disinfecting, and ethylene glycol for the preservation of tissue plasticity, whereas the concentration of formalin is kept to a strict minimum (0.8%) [4]. This technique helped in the preservation of color, consistency, and transparency of the tissue with no harmful substances released. Dräger capillaries do not detect formaldehyde in room air. Bacteriologic tests were used to detect the efficacy of disinfectants. No mold growth was detected in any cadaver [3].

We selected one donated body and three unclaimed bodies of destitute patients for soft embalming after proper clearance from authorities. We followed the technique and chemicals described by Reddy et al. [5] in their studies. Due to unavailability at our place, we replaced 4‑chloro‑3‑methylphenol 1% with phenol 1%. The fluid for embalming was prepared using two stem solutions as the base:

Stem solution A - boric acid 3%: 9 kg; (mono)ethylene glycol 30%: 19 L; ammonium nitrate 20%: 12.6 kg; potassium nitrate 5%: 3.2 kg; water: 63.3 L; total: 100 L

Stem solution B - (mono)ethylene glycol 10%: 18.2 L; phenol 1%: 1.8 kg; total: 20 L

Embalming solution - stem solution A: 14.3 L; stem solution B: 0.5 L; formalin: 0.3 L; sodium sulfite: 0.7 kg

Total: 15.8 L

Sodium sulfite and formalin are added just before perfusion. The final solution had <0.5% formaldehyde. The cadavers are perfused through the great saphenous vein if found or through the femoral or carotid artery. A total of 14 liters of the embalming solution is perfused per cadaver. The cadavers were kept in refrigerators at 40°C for preservation before being used for the dissection purpose. The cadavers were used to teach pedicle screw instrumentation to delegates registered for the workshop. Feedback questionnaires (Table 1) were provided to the participants (n=28), consisting of both faculty members and delegates, for rating the quality of the soft embalming of the provided cadavers on various aspects. The questionnaires had 14 items. Besides asking about the status of their designation (delegate or faculty member of a medical college) and their experience of exposure to any type of cadaver embalmed surgical skill training (both formalin embalmed cadavers and specifically soft embalmed cadavers), it asked questions that measured the suitability of the soft embalmed cadavers on four domains for surgical skill learning (Table 2), for example, surgical suitability, X-ray visualization, lack of unpleasant smell and mucous irritation, and the color of the skin. Each of the domains was measured by the items (on a scale of 1 to 4), as elaborated in Table 1.

**Table 1:** Questionnaire

Question number	
1	What is your role in the program?
	(a) Faculty member: 1
	(b) Delegate: 2
2	Do you have any previous experience with cadaver hands-on program? If yes, please mention the program and the procedure demonstrated or practiced
	(a) Yes: 1
	(b) No: 2
3	Do you have any previous experience with soft embalmed cadavers? If yes, please mention the program and the procedure demonstrated or practiced
	(a) Yes: 1
	(b) No: 2
4	Which of the following hold true for a normal formalin-fixed cadaver for you?
	(a) The color doesn’t appear life-like: 1
	(b) The tissue texture is too hard for dissection: 2
	(c) The body has a foul smell: 3
	(d) Mucosal irritation is unbearable sometimes: 4
	(e) Instrumentation is difficult: 5
	(f) Intra-procedure imaging is of poor quality: 6
5	Did you experience any foul smells during today’s workshop?
	(a) Yes, and it was unbearable: 1
	(b) Yes, but I can tolerate it: 2
	(c) Yes, very mild: 3
	(d) No smell at all: 4
6	Did you feel any mucosal irritation during today’s workshop?
	(a) Yes, and it was unbearable: 1
	(b) Yes, but I can tolerate it: 2
	(c) Yes, very mild: 3
	(d) No irritation at all: 4
7	How was the color of the body?
	(a) It was like normal human skin and body color: 4
	(b) It was like normal human skin but not the same: 3
	(c) It was not like normal human skin: 2
	(d) It was black and discolored: 1
8	How was the texture and pliability of the tissue?
	(a) Soft and like living human beings: 4
	(b) Little bit hard but still pliable: 3
	(c) Tissue was hard but can be cut easily: 2
	(d) Tissue was very hard and dry. Difficult to cut: 1
9	Was proper positioning possible for the instrumentation?
	(a) Yes, positioning can be achieved, and the joints were supple: 4
	(b) Yes, positioning could be achieved but the joints were not supple: 3
	(c) Yes, positioning could be achieved with difficulty: 2
	(d) No, it was not possible to achieve proper positioning for instrumentation: 1
10	Was it useful for learning surgical exposure?
	(a) Extremely useful: 4
	(b) Very useful but better than formalin-fixed cadaver: 3
	(c) As useful as while using normal formalin-fixed cadaver: 2
	(d) Not at all useful: 1
11	How were the anatomical features and tissue planes?
	(a) Extremely well preserved and discernible tissue planes: 4
	(b) Very well preserved but tissue planes were somewhat difficult to differentiate: 3
	(c) Well preserved but tissue planes couldn’t be found properly: 2
	(d) Anatomical features were not preserved: 1
12	Was it useful for using surgical instruments and screw insertion?
	(a) Extremely useful: 4
	(b) Very useful: 3
	(c) Somewhat useful: 2
	(d) Not useful at all: 1
13	Was it suitable for imaging studies?
	(a) Extremely useful: 4
	(b) Very useful: 3
	(c) Somewhat useful: 2
	(d) Not useful at all: 1
14	How were the X-ray images taken while inserting pedicle screw and instrumentation?
	(a) Extremely good quality and clarity: 4
	(b) Very good quality and clarity: 3
	(c) Somewhat good but blurred or obscured with shadows: 2
	(d) Not good at all: 1

**Table 2:** Domains for surgical skill learning

Domain	Items
Surgical suitability	8,9,10,11,12
X-ray visualization suitability	13,14
Lack of unpleasant smell and mucous irritation	5,6
Color of the skin in the embalmed cadaver	7

After completion of the workshop, the bodies were again vacuum wrapped in polythene and kept in the refrigerator at 40°C. The bodies were seen at regular intervals to document the changes found. No further studies were conducted.

## Results

After the data were compiled and arranged in a tabular form in Windows Excel, it was converted into a .CSV file and processed in a statistical program JASP Version 0.17 [6]. The descriptive statistics showed seven missing values. The details of the descriptive statistics are given in Table 1. The total number of participants was 28, of whom 14 (50%) were faculty members and 14 (50%) were delegates. Among the participants, 68% (n=19) were exposed to some form of cadaver surgical training (either formalin preserved or soft embalming), and 54% (n=15) were specifically exposed to surgical skill training involving soft-embalmed cadavers. In response to item 5, 64% of the participants confirmed that the cadavers did not emit any smell at all. In response to item number 6, 82% of the participants were of the view that there was no nasal and eye irritation (which usually occurs in working with formalin-preserved cadavers). Because many participants were exposed to both forms of embalmed cadavers (Formalin vis-à-vis soft embalmed cadavers), they could easily discern the comfort of working with soft-embalmed cadavers. In response to item 7, 64 % (n=18) of participants were of the view that the color of the cadavers resembled that of a live human being but not the same; 25% (n=7) of participants thought the color of the prepared cadavers resembled the skin tone of live human beings. Therefore, 89% (n=25) of the participants agreed that the skin tone of the prepared cadavers resembled that of a live human being. This makes surgical skill training more realistic. In response to item 8 (Table 2), 46% (n=13) opined that the tissues were soft and pliable like that of a live anesthetized patient, whereas 50% (n= 14) thought that the tissue was soft and pliable albeit a little hard as compared to a live patient. Therefore, 96% (n=27) of participants believed that the tissue was broadly soft and pliable, which is an important characteristic to be achieved in any soft-embalming preparation; 93% (n=26) of participants agreed that positioning the cadavers could be easily achieved (an important characteristic in any cadaver-surgical training) and that the suppleness of the joints was adequate. Also, 82% (n=23) of the participants agreed that the prepared soft-embalmed cadavers was useful for learning surgical exposure, 54% (n=15) of participants thought they can easily differentiate between different anatomical planes, at the same time, and 35% (n=10) of participants thought that they had a little difficulty in distinguishing different anatomical planes. Again, 72% (n=20) of the participants thought that the training in instrumentation and pedicle screw insertion in the prepared cadavers were useful. Regarding imaging suitability (item no 13, Table 2), 53% (n=15) of the participants thought that the image quality of the CT scans and X-ray of the prepared cadavers were “extremely useful”; at the same time, 40% (n=11) thought that the above images were “very useful.” Visualization of the cervical vertebral bones during cadaver surgical training in the C-arm monitor was rated to be of “extremely good clarity” by 40% (n=11) of participants, whereas an equal number of participants thought it to be of “very good clarity.”

When scores of item 8 to item 12 for each participant were evaluated for internal reliability by estimating its Cronbach α at a 95% confidence interval, the α-value turned out to be “good” (α=0.827). When item 13 and item 14 were subjected to the above test, Cronbach α turned out to be “acceptable” (α=0.739) [7,6]. When the scores of item 5 and item 6 were examined for internal reliability by determining Cronbach α, they turned out to be “good” (α=0.822). The authors are of the view that the scores of items 8 to 12, items 13 to 14, and items 5 to 6 measure three different aspects or domains measuring the quality of soft-embalmed cadaver preservation. The authors are aware that to substantiate that these scores are measuring a particular domain of “quality of soft-cadaver preservation,” exploratory factor analysis and principal component analysis are required. The authors intend to name the domains being measured surgical suitability (scores of items 8 to 12), imaging suitability (scores of items 5 and 6), and smell score (scores of items 5 and 6). It can be a guide to construct and refine a better quantitative scale to measure the “quality of soft-embalmed cadavers for surgical training.”

We took swabs from the nasal, oral, and anal regions from three out of four Thiel embalmed cadavers in our study. Fluid was collected from the thoracic and abdominal cavities using a sterile technique. The first corpse showed *Candida* growth from nasal swabs. The second corpse showed *Enterococcus* sp. growth from oral and anal swabs. The third cadaver showed *Micrococcus* growth from nasal, oral, and anal swabs, *Enterococcus* growth from oral and anal swabs, and *Candida *growth from nasal and oral swabs. None of the cadavers showed any growth from fluid taken from thoracic or abdominal cavities (Table 3).

**Table 3:** Culture from cadavers

No	Nasal swab	Oral swab	Anal swab	Thoracic cavity fluid	Abdominal cavity fluid
1	*Candida *Sp	No growth	No growth	No growth	No growth
2	No growth	*Enterococcus* Sp	*Enterococcus *Sp	No growth	No growth
3	*Micrococcus *and *Candida *Sp	*Micrococcus, Enterococcus *and *Candida *Sp	Micrococcus, Enterococcus	No growth	No growth
4	Not taken	Not taken	Not taken	Not taken	Not taken

## Discussion

The formalin-fixed bodies pose a health hazard and are not suitable for fine dissection due to overhardening. [8] Exposure to formaldehyde is associated with the risk of carcinogenesis. [9] Common symptoms such as fatigue and sensation of burning in the eyes and nose were found in those exposed to formalin during cadaver dissection [10]. The soft tissue textures, anatomy, and tissue planes are better discernible in Thiel embalmed cadavers than in normal formalin-fixed cadavers. The better flexibility of a soft embalmed cadaver can help in the better detection of tissue planes during dissection [11]. Nowadays, a new embalming technique using N-vinyl-2-pyrrolidone has been used in cadaver dissection to teach procedures such as endoscopic transnasal skull base approach, laparoscopic training, endotracheal intubation, and motion physiology of vocal folds [8,12,13].

In our study, all the participants were inquired about their earlier experience with cadaver dissection. All the faculties had earlier experience with soft embalmed cadavers, whereas a few delegates had any earlier exposure. Most of the participants had a better experience with the soft embalmed cadavers in terms of better texture, body color, tissue planes, odor, suppleness of joints with ease of positioning, and X-ray imaging, which has a vital role in pedicle screw insertion. The participants had faced difficulties with formalin-fixed cadavers in their earlier experience.

Another important risk of dissection is the exposure of the dissectors to various microorganisms. When a person is alive, various natural surfaces and cavities of the body such as skin, gastrointestinal tract, genitourinary tract, respiratory tracts, and oral cavity host multiple microorganisms [14,15]. Various estimates suggest that a human's ratio of microflora to body cells can range from 1.3 times [16] to 10 times [17]. When the host is alive, these microorganisms help in the normal biological activities of the body, such as immunogenesis and nutritional supplementation [18]. Once the host dies, the autolysis starts with enzymatic degradation of various body tissues. Afterward, those organisms living in the gastrointestinal, urogenital tract, and so on help in breaking the various elements in the body, which results in decay or putrefaction after death [19-21].

Therefore, the embalming technique used should have a good disinfectant property to save the persons exposed to the cadavers from various infections. In a study comparing four different methods of embalming using Thiel, formalin, Genelyn and the Imperial College London soft preservation (ICL-SP) solution to evaluate the disinfectant property of the chemicals used Balta et al. found that as compared to the growth obtained before embalming formalin and Genelyn embalmed cadavers showed no growth after embalming, Thiel-embalmed cadavers showed significantly less number of colonies, while the number of colonies remained almost the same in ICL-SP-embalmed cadavers. Therefore, it was concluded that formalin-embalmed cadavers showed the strongest disinfecting abilities followed by Thiel-embalmed cadavers, then Genelyn-embalmed cadavers, and, finally, ICL-SP cadavers [22].

Our study considered spine surgeons, but the same principle applies to all specialty training [23]. First, we have tried to develop an embalming solution as per the resources available at our center or region by modifying an index solution. The characteristics of the soft embalmed cadavers prepared using our solution were satisfactory. Those soft embalmed cadavers can be preserved in refrigerators and can be used repetitively to study different parts of the body. The authors have tried to define various domains to assess the quality of soft embalmed cadavers that can be prepared in other centers for such training purposes.

Our study limitation was that it is a single-center study and has only considered spine surgeons.

## Conclusions

Skill laboratories give opportunities to young surgeons and trainees to learn and improve their skills before applying them to real patients. This was our first attempt to develop soft embalmed cadavers at our center and our state. We used the parent solution with some variations as per the availability of chemicals at our place and found that the features of the preserved cadavers were good and well-suited to address our purpose. Therefore, with some variations in the parent formulations, centers situated in remote and less developed areas can formulate their own solution to develop soft embalmed cadavers and establish cadaver skill laboratories. This will benefit the local surgeons and trainees. The authors tried to develop a few domains through statistical analysis, which can be used to assess and compare the quality of cadavers prepared at various centers.

## References

[1] Serageldin I (2013). Ancient Alexandria and the dawn of medical science. Glob Cardiol Sci Pract.

[2] Elizondo-Omaña RE, Guzmán-López S, García-Rodríguez Mde L (2005). Dissection as a teaching tool: past, present, and future. Anat Rec B New Anat.

[3] Thiel W (1992). [The preservation of the whole corpse with natural color] [Article in German]. Ann Anat.

[4] Groscurth P, Eggli P, Kapfhammer J, Rager G, Hornung JP, Fasel JD (2001). Gross anatomy in the surgical curriculum in Switzerland: improved cadaver preservation, anatomical models, and course development. Anat Rec.

[5] Reddy R, Iyer S, Pillay M, Thankappan K, Ramu J (2017). Soft embalming of cadavers for training purposes: Optimising for long-term use in tropical weather. Indian J Plast Surg.

[6] JASP Team (2023 (2023). JASP Team. JASP (Version.

[7] George D, Mallery P (2010). SPSS for windows step by step: a simple guide and reference 18.0 update. Upper Saddle River, NJ: Pearson.

[8] Nagase M, Nagase T, Tokumine J, Saito K, Sunami E, Shiokawa Y, Matsumura G (2022). Formalin-free soft embalming of human cadavers using N-vinyl-2-pyrrolidone: perspectives for cadaver surgical training and medical device development. Anat Sci Int.

[9] Adamović D, Čepić Z, Adamović S, Stošić M, Obrovski B, Morača S, Vojinović Miloradov M (2021). Occupational exposure to formaldehyde and cancer risk assessment in an anatomy laboratory. Int J Environ Res Public Health.

[10] Soonklang N, Saowakon N (2022). Evaluation of formaldehyde exposure among gross dissection after modified embalming solution and health assessment. Environ Sci Pollut Res Int.

[11] Kennel L, Martin DM, Shaw H, Wilkinson T (2018). Learning anatomy through Thiel- vs. formalin-embalmed cadavers: student perceptions of embalming methods and effect on functional anatomy knowledge. Anat Sci Educ.

[12] Maruyama K, Yokoi H, Nagase M (2019). Usefulness of N-vinyl-2-pyrrolidone embalming for endoscopic transnasal skull base approach in cadaver dissection. Neurol Med Chir (Tokyo).

[13] Nagase M, Kimoto Y, Sunami E, Matsumura G (2020). A new human cadaver model for laparoscopic training using N-vinyl-2-pyrrolidone: a feasibility study. Anat Sci Int.

[14] Costello EK, Lauber CL, Hamady M, Fierer N, Gordon JI, Knight R (2009). Bacterial community variation in human body habitats across space and time. Science.

[15] Luczynski P, McVey Neufeld KA, Oriach CS, Clarke G, Dinan TG, Cryan JF (2016). Growing up in a bubble: using germ-free animals to assess the influence of the gut microbiota on brain and behavior. Int J Neuropsychopharmacol.

[16] Sender R, Fuchs S, Milo R (2016). Are we really vastly outnumbered? Revisiting the ratio of bacterial to host cells in humans. Cell.

[17] Blaser MJ, Webb GF (2014). Host demise as a beneficial function of indigenous microbiota in human hosts. mBio.

[18] Wilson M (2008). Bacteriology of Humans: An Ecological Perspective. https://www.wiley.com/en-us/Bacteriology+of+Humans%3A+An+Ecological+Perspective-p-9781405161657.

[19] Vass AA, Barshick SA, Sega G, Caton J, Skeen JT, Love JC, Synstelien JA (2002). Decomposition chemistry of human remains: a new methodology for determining the postmortem interval. J Forensic Sci.

[20] Dent BB, Forbes SL, Stuart BH (2004). Review of human decomposition processes in soil. Environ Geol.

[21] Evans WED (1963). The Chemistry of Death.

[22] Balta JY, Cryan JF, O'Mahony SM (2019). The antimicrobial capacity of embalming solutions: a comparative study. J Appl Microbiol.

[23] Asghar A, Naaz S, Patra A, Ravi KS, Khanal L (2022). Effectiveness of 3D-printed models prepared from radiological data for anatomy education: a meta-analysis and trial sequential analysis of 22 randomized, controlled, crossover trials. J Educ Health Promot.

